# Fragility of L5 Vertebral Fracture After Rod Fracture at the Lumbosacral Junction Following Long-Segment Spinal Fusion Surgery for Adult Spine Deformity

**DOI:** 10.7759/cureus.43242

**Published:** 2023-08-09

**Authors:** Shun Okuwaki, Toru Funayama, Kengo Fujii, Masaki Tatsumura, Masashi Yamazaki

**Affiliations:** 1 Department of Orthopaedic Surgery, Kenpoku Medical Center Takahagi Kyodo Hospital, Takahagi, JPN; 2 Department of Orthopaedic Surgery, University of Tsukuba, Tsukuba, JPN; 3 Department of Orthopaedic Surgery, Showa General Hospital, Kodaira, JPN; 4 Department of Orthopaedic Surgery and Sports Medicine, Tsukuba University Hospital Mito Clinical Education and Training Center, Mito Kyodo General Hospital, Mito, JPN

**Keywords:** fragility fracture, hyperextension vertebral fracture, lumbosacral junction, rod fracture, adult spinal deformity

## Abstract

We report a case of vertebral fracture in a patient with rod fractures after adult spinal deformity surgery, which occurred at the same level as the rod fractures, even though intervertebral bone fusion in the fusion range had been achieved.

A 77-year-old female underwent corrective spinal surgery for adult spinal deformity from T12 to the pelvis but had a subsequent uppermost instrumented vertebral fracture, resulting in pseudarthrosis and severe kyphosis. The patient underwent proximal fusion extension to the T4, which improved alignment. A right-sided rod fracture at the lumbosacral junction occurred after 18 months; however, it showed no symptoms. After a month, the patient experienced severe low back pain with left leg pain and was diagnosed with bilateral rod fractures associated with L5 hyperextension vertebral fracture. The patient underwent revision surgery to repair the fractured rods with a multiple-rod construct.

Rod fractures can occur even when bone fusion is achieved within the fusion range. When rod fractures are detected at the lumbosacral junction even if the interbody fusion was achieved, a hyperextension vertebral fracture may occur.

## Introduction

Rod fracture (RF) is a frequent implant-related complication, with a high incidence (range, 6.8%-22%), after corrective fusion surgery for adult spinal deformity (ASD) [1-8]. RF after long-segment corrective fusion surgery is often caused by residual instability without intervertebral bony fusion within the fusion range, resulting in an increased load on the rods. Pseudarthrosis is a particularly prevalent complication that most commonly occurs at the three-column osteotomy site or the lumbosacral junction [9, 10]. When complete interbody fusion is achieved and the risk of RF is low. And when the RF is asymptomatic, the patient can be followed up without a revision surgery [11]. Few cases of vertebral fracture resulting from RF after corrective fusion surgery for ASD have been reported.

Herein, we report a case of fragility vertebral fracture in a patient with RFs after ASD surgery, which occurred at the same spinal level as the RFs, even though interbody fusion was successfully achieved.

## Case presentation

**Figure 1:**
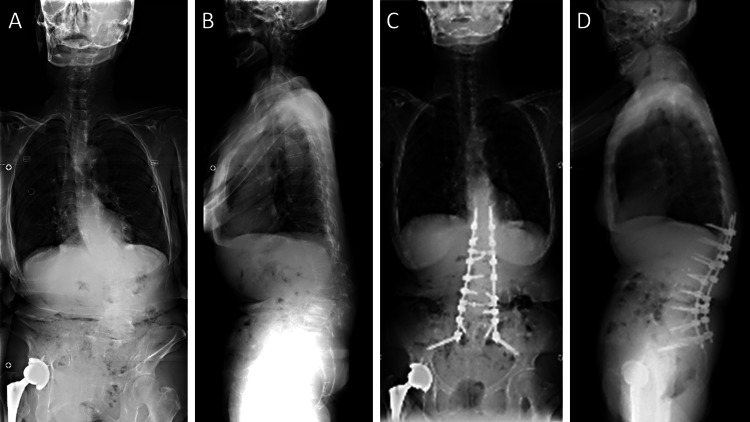
Preoperative and postoperative images of the initial surgery. (A, B) The patient was diagnosed with adult spinal deformity at another hospital 7 years ago. (C, D) Immediately after the corrective fusion surgery from T12 to the pelvis, the patient developed an uppermost instrumented vertebral fracture.

A 77-year-old female underwent posterior corrective fusion from T12 to the pelvis with multiple interbody fusions as the initial surgery for ASD at another hospital seven years ago. Immediately after the surgery, the patient developed an uppermost instrumented vertebral fracture (Figure 1).

**Figure 2:**
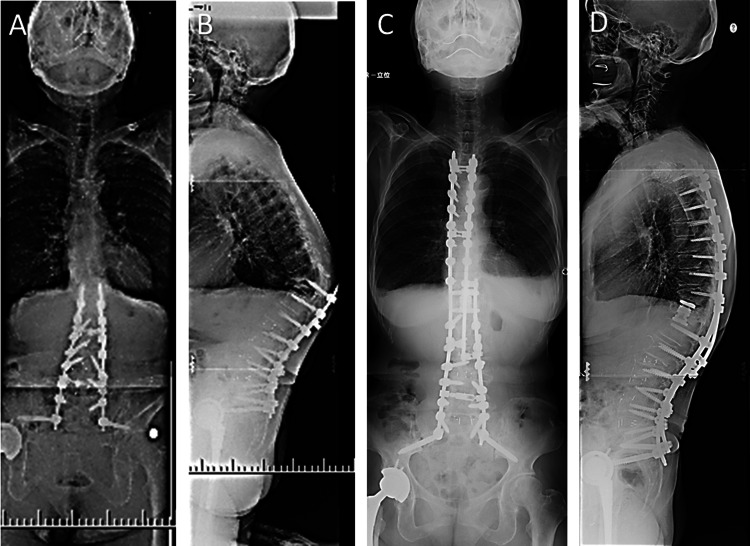
Preoperative and postoperative images of the second surgery. (A, B) The patient presented with pseudarthrosis of T12 and resultant severe kyphosis when referred to our hospital 3 years ago. (C, D) The patient underwent the second surgery to extend the fusion to T4 with T12 corpectomy.

The patient was referred to our hospital three years ago and presented with pseudarthrosis of T12 and resultant severe kyphosis. Radiographic alignment was as follows: sagittal vertical axis, 65 mm; lumbar lordosis, 32°; pelvic incidence, 65°. The patient underwent the second surgery to extend the fusion to T4 with T12 corpectomy. The patient, fused with a dual-rod construct to the pelvis, received a single rod on each side of the spinal column fixed to the pelvis using bilateral S2 alar-iliac (SAI) screws and S1 pedicle screws. Cobalt chrome rods, with 5.5 mm in diameter, were used and the patient received a bone grafting at the surgical site including lumbosacral joint with local autograft bone and unidirectional porous β-tricalcium phosphate, mixed in approximately 1:1 ratio [12] (Figure 2).

**Figure 3:**
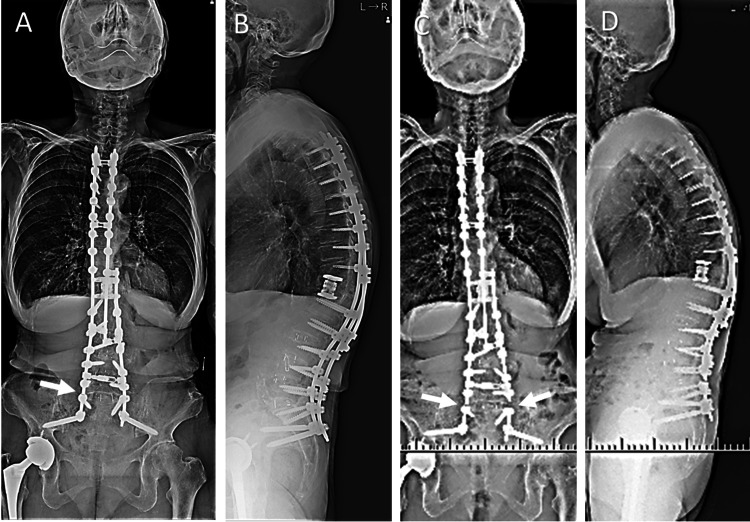
Follow-up images after the second surgery. (A, B) Eighteen months after the second surgery, right-sided RF was incidentally detected on radiographs (arrow) without alignment changes or symptoms. (C, D) Six months after the unilateral RF, bilateral RFs (arrows) occurred with severe left leg pain. RF: rod fracture

After 18 months, right-sided RF was incidentally detected on radiographs at a follow-up visit. No alignment changes or symptoms were observed. Six months after the unilateral RF, severe left leg pain developed from the buttock to the thigh when lifting an object. Radiographs showed bilateral RFs (Figure 3).

**Figure 4:**
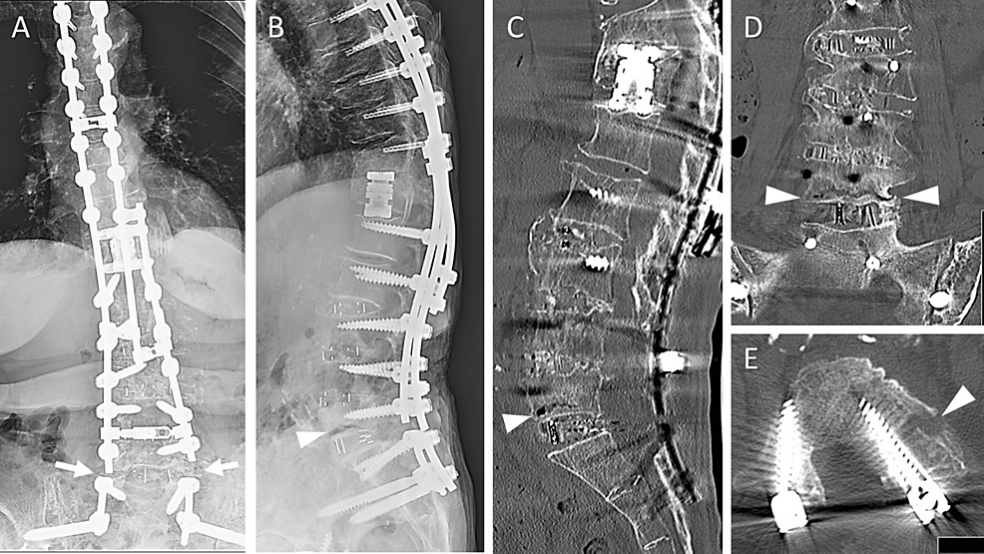
Images of hyperextension vertebral fracture at L5 after bilateral rod fractures. (A, B) Radiographs showed bilateral rod fractures (arrows) and a hyperextension vertebral fracture at L5 (arrowhead). (C, D) CT showed that the vertebral fracture has occurred at the caudal side of the verterbral body (arrowheads). On the other hand, complete interbody fusions at L4-L5 and L5-S1 were detected. (E) CT showed the displaced left pedicle fracture at L5 (arrowhead). CT: computed tomography

Computed tomography (CT) revealed complete interbody fusions at L4-L5 and L5-S1 without pedicle screw loosening; however, the non-displaced left pedicle fracture at L5 was detected (not shown). The patient was diagnosed with left L5 radiculopathy due to the pedicle fracture and was scheduled for revision surgery. However, the low back and lower extremity pain increased shortly after that, and the patient experienced difficulty moving due to the pain. Plain radiographs and CT showed a hyperextension vertebral fracture at L5 with a displaced left pedicle fracture (Figure 4). Before, the patient was used on teriparatide, and dual-energy X-ray absorptiometry of the left total hip showed a bone mineral density of 0.955g/cm^2 ^and a T score of 0.2. Subsequently converted to romosozumab with the expectation of promoting bone formation.

**Figure 5:**
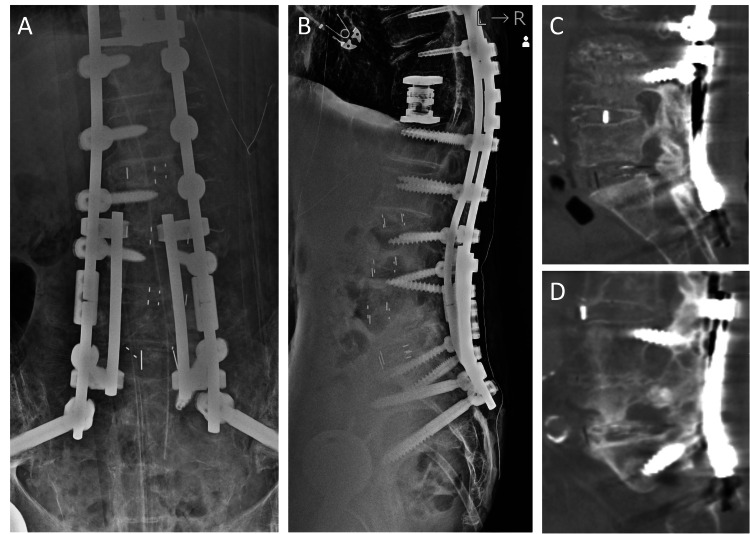
Postoperative images of the third surgery. (A, B) After the pedicle screws at L5 were removed, the fractured rods were repaired with axial connectors. The additional rods were fixed from L3-L4 to S1-SAI. (C, D) Bone fusion of the grafted bone of the posterior element was achieved. SAI: S2 alar-iliac

The patient underwent the third surgery. After the pedicle screws at L5 were removed, the fractured rods were repaired with axial connectors without a decompression procedure. The additional rods were fixed from L3-L4 to S1-SAI. The patient received a local autograft bone and demineralized bone matrix (Figure 5).

**Figure 6:**
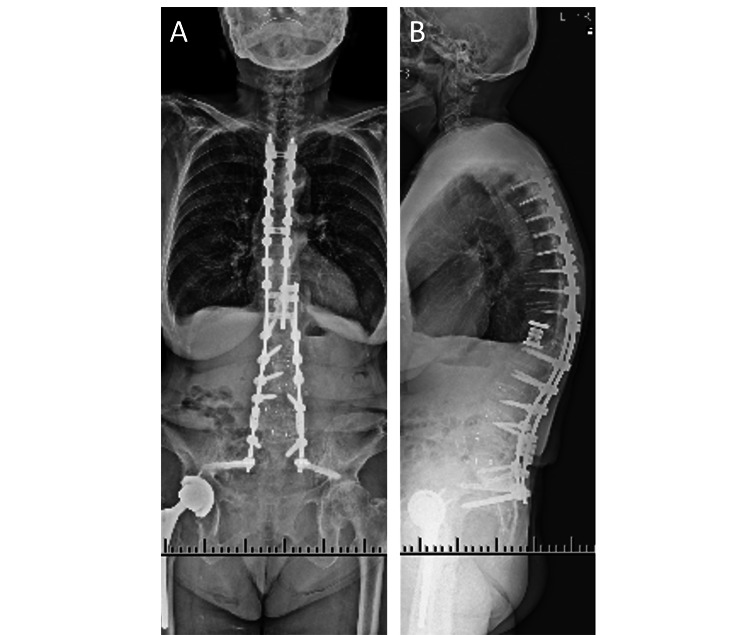
Final follow-up images. (A,B) After 2-year revision surgery, radiographs showed bone fusion at L5, and alignment was maintained.

Immediately, the patient's low back and left leg pain improved. However, surgical site infections were complicated postoperatively. The additional rods were removed because the surrounding tissue of the additional rods was thought to be the origin of the infection. Debridement and continuous local antibiotic perfusion using aminoglycoside (gentamicin), and a negative pressure wound therapy [13] were performed. In addition, a long-term antibiotics was administered, and the infection was successfully controlled. At the two-year postoperative follow-up, a solid bony fusion of the L5 vertebral body and posterior element at the lumbosacral joint was achieved, resulting in apparent pain relief (Figure 6).

## Discussion

Herein, we present a rare case of a fragility vertebral fracture despite of successful interbody fusion after corrective fusion surgery for ASD. In the present case, the L5-S1 intervertebral level lacked a posterior bony structure after posterior interbody fusion at the initial surgery, and there was subjected to long-segment fusion from the upper thoracic spine to the pelvis. This led to bilateral RFs at the L5-S1 intervertebral level owing to the mechanical stress from the long lever arm. In addition, the L5 vertebral body experienced concentrated stress due to the absence of posterior bony support after the bilateral RFs, resulting in a hyperextension vertebral fracture with a three-column injury [14,15] with a unilateral pedicle fracture. Since there were no symptoms at the time of the initial rod fracture, we consider that the vertebral fracture was secondary to the initial rod fracture.

RFs due to pseudarthrosis after interbody fusion surgery frequently occurs at the lumbosacral junction, and the lumbosacral pseudarthrosis rate with the dual-rod is consistent with a range of 18.5-26.0% [9, 10, 16, 17]. In previous reports, high biomechanical loads and shear forces make the lumbosacral junction particularly difficult to stabilize during fusion [18-20]. The longer the fusion construct, the higher the incidence of lumbosacral pseudarthrosis, suggesting that increased mechanical forces contribute to nonunion [21]. In the present case, bony fusion was achieved at the lumbosacral junction by proper anchoring to the pelvis. Rod repair is considered when alignment changes occur after RFs. Yamato et al. reported that a revision surgery should be indicated when loss of deformity correction and alignment deterioration are detected [11]. In the present case, bilateral RFs led to a vertebral fracture within a short period.

A factor that may have contributed to the fracture is the confirmed effect of stress shielding on spinal instrumentation even after interbody fusion was achieved [22]. In addition, long fusion constructs increase the lever arm and moment at the lumbosacral junction. A previous report showed that L5 fractures are more common in cases of implant removal after long spinal fusion [23]. Because reinforcement was not performed due to the RFs, a hyperextension vertebral fracture occurred at L5, and neurological symptoms were also observed. The present case demonstrated that RFs could occur even though interbody fusion was achieved. Therefore, it is necessary to actively consider reinforcement if RFs occur, even if image-based bony fusion is achieved.

Rigid surgical instrumentation is required to stabilize the lumbosacral junction when implant devices are used to prevent RFs after pseudarthrosis. Multi-rod constructs have shown increased biomechanical stability than canonical dual-rod constructs; mechanical instability causes lumbosacral pseudarthrosis, and the increased stability of multiple rods will improve fusion rates [24-27].

## Conclusions

In conclusion, RFs can occur even when bone fusion is achieved within the fusion range. When RFs are detected at the lumbosacral junction even if the interbody fusion was achieved, a hyperextension vertebral fracture, such as that described in this case, may occur. Therefore, careful follow-up is recommended if a RF occurs.
